# Changes in Psychosocial Variables Among Caregivers of Patients With Schizophrenia: A Short-Term Follow-Up Study

**DOI:** 10.7759/cureus.55887

**Published:** 2024-03-10

**Authors:** Sanimar S Kochhar, Ashwani K Mishra, Rakesh K Chadda, Mamta Sood, Rachna Bhargava

**Affiliations:** 1 Clinical Psychology, All India Institute of Medical Sciences, New Delhi, New Delhi, IND; 2 Biostatistics, All India Institute of Medical Sciences, New Delhi, New Delhi, IND; 3 Psychiatry, Amrita Hospital, New Delhi, IND; 4 Psychiatry, AIl India Institute of Medical Sciences, New Delhi, New Delhi, IND

**Keywords:** family functioning, coping, mental illlness, longitudinal, experience of caregiving, schizophrenia, caregivers

## Abstract

Background and aims: Caring for a person with schizophrenia entails considerable effort. The caregiving experience while caring for a person with schizophrenia has been described as both stressful and enriching. The literature on changes in caregiving experience with time has been fairly limited. The present analysis looks at the change in the caregiving experience of caregivers of patients with schizophrenia.

Method: This study had a sample of 50 caregivers of patients with schizophrenia who were followed up after a period of 6 to 10 months. Caregiving experience, knowledge of the mental illness, family functioning, coping, social support, psychological distress, quality of life, and spiritual, religious, and personal beliefs were assessed at the two time points.

Results: Among the negative caregiving experiences, caregivers' need for back-up and patient dependency reduced significantly at follow-up as compared to baseline, though total negative or positive experiences of caregiving did not show a significant difference. Knowledge about mental illness did not significantly increase at follow-up, though issues related to poor communication and affective involvement in family functioning reduced over the period of time. Coping and social support remained largely the same, while psychological distress was reduced. All domains of quality of life improved over the follow-up period, while the hope, optimism, and inner peace aspects of spiritual, religious, and personal beliefs improved with time.

Conclusion: Some aspects of caregiving experiences may change with time. As caregivers become more adjusted to caregiving roles, their distress may reduce, and their quality of life may improve.

## Introduction

Schizophrenia is an important psychiatric illness that impacts not only the patient but also family members and caregivers. These impacts include medication costs and non-adherence, handling bizarre behaviour and violence, substantial caregiving tasks, family conflict, stigma, and discrimination [1]. There are also emotional responses felt by caregivers, such as shame, guilt, worry, fear, sadness, and loss [2]. However, greater research is required to consider the possibility of positive growth and well-being connected to caregiving [3]. Caregivers are not only expected to facilitate the treatment of the patient suffering from schizophrenia but also to endure the psychiatric symptoms; to take care of health, hygiene, and dressing; to enable socialization and livelihood; and to provide social support [4]. Caregivers have a considerable role in determining how patients with schizophrenia encounter and interact with the world, i.e., their role performance [5], thereby modulating the outcomes of the individual [2]. Caregivers have often been roped in to help with the day-to-day functioning of patients suffering from schizophrenia [6].

Caregiving has been considered stressful, affecting the caregivers in various ways. The burden among caregivers of patients with schizophrenia has been reported in several studies, including reviews [7,8]. Challenges associated with the caregiving role have been expressed as physical and psychological symptoms, changes to the quality of life, and changes in the outlook towards circumstances. Many caregivers also experience proxy stigma due to the caregiving role that they play. Nonetheless, caregiving for patients with schizophrenia is not necessarily a negative experience. Several caregivers appraise certain positive aspects of caregiving, such as positive personal experiences resulting from caregiving and the good aspects of the patient-caregiver relationship [9]. Also, the caregiving process has been acknowledged as satisfying and stimulating one’s growth by some [10]. Thus, caring for a person with schizophrenia in the family context has many intertwined facets. The caregiving experience is also associated with other factors like family functioning, caregiver coping, social support, and psychological distress, as per the extant literature [9,11].

While cross-sectional assessments of caregiving experience have been carried out in the studies mentioned above [9,11], the change in caregiving experience has extremely limited literature. A systematic review by Karambelas et al. [3] also emphatically remarks that studies should be conducted on changes in caregiving experiences with time. While caregiver burden and distress have been studied in a longitudinal manner [12], caregiving experience has not been studied longitudinally among caregivers of schizophrenia. Understanding such changes in caregiving experience can help to discern what aspects change with time and to what extent. The present study looks at the changes in experiences of caregiving for persons with schizophrenia over a period of follow-up.

## Materials and methods

Setting and participants

This study was conducted at a tertiary care hospital and its public-funded medical school in India. A significant proportion of patients seeking help in psychiatry services are those with schizophrenia and belong to all strata.

Sample

The sample was drawn from the outpatient services of the Department of Psychiatry. Purposive sampling was used to constitute the sample (n = 50) with predetermined inclusion and exclusion criteria. Patients aged 18 to 55 years, of either gender, with a diagnosis of schizophrenia as per ICD-10 criteria of more than one-year duration were included. Caregivers were included if they lived with the patient for more than a year, were mainly involved in caring for the patient and supervising the treatment (including visits to the hospital), and were able to read Hindi, the local language. Exclusion criteria for caregivers were disabling physical or psychiatric disorders, having another family member with a diagnosed disabling chronic medical illness or comorbid disabling psychiatric disorder staying in-house, and patients with comorbid and disabling chronic physical or psychiatric disorders, substance dependence (except tobacco), or organic brain syndromes.

Ethical considerations

Ethical approval was obtained from the Institute Ethics Committee, AIIMS, New Delhi (Approval Number: IECPG-204/10.05.2018).

Procedure

The study was initiated after ethical clearance from the Institute Ethics Committee. Patients and caregivers were recruited after obtaining informed consent. They were assessed for socio-demographic and clinical variables. The caregivers assessed for caregiving experiences using the Experience of Caregiving Inventory [13]. In them, knowledge was assessed using the Knowledge of Mental Illness (KMI) Scale [14]; the Family Assessment Device (FAD) [15] assessed family functioning; the Coping Checklist [16] assessed coping; the Social Support Questionnaire [17] measured social support; the General Health Questionnaire-12 [18] measured psychological distress; the World Health Organization Quality of Life Instrument (WHOQOL)-BREF [19] measured the quality of life; and the World Health Organization quality of life Spirituality, Religiosity and Personal Beliefs Instrument (WHOQOL-SRPB) [20] measured spirituality, religiosity, and personal beliefs. The cases were re-assessed after 6-10 months on the same psychosocial variables. Thus, data for those 50 caregivers who had completed the baseline and second assessments were analyzed.

Measures

The Experience of Caregiving Inventory is a 66-item self-report scale that assesses both positive and negative aspects of caregiving. Each of the questions is rated on a 5-point Likert scale. The scale has ten dimensions: eight negative and two positive. Total negative and positive domain scores are also calculated. The subscale Cronbach alphas range from 0.74 for dependency to 0.91 for difficult behaviours [13].

The KMI Scale assesses knowledge about various aspects of mental illness, such as diagnosis, symptoms, causes, medication, and treatment. It comprises five items rated on a 2-point scale. Higher scores indicate better knowledge of the illness [14].

The Family Assessment Device (FAD) comprises 60 items that are self-reported on a 4-point scale. Higher scores mean poorer family functioning. It has seven dimensions that examine various aspects of family functioning. Cut-offs for identifying healthy and unhealthy families exist. Cronbach’s alphas in the various domains range from 0.72 to 0.92 [15].

The Coping Checklist [16] is a 70-item questionnaire that covers the coping methods utilized to cope with stress. It has seven subscales that encompass problem-focused and emotion-focused coping components. The total score for each subscale is produced by summing the number of responses. The internal consistency of this scale has been seen as adequate (full scale: alpha = 0.86). The test-retest reliability for one month has been found to be 0.74.

The Social Support Questionnaire [17] consists of 18 items on a 4-point Likert scale. A higher score indicates greater social support. The test-retest reliability of the modified version of the SSQ is high (correlation coefficient = 0.91, p<0.01).

The General Health Questionnaire-12 (Hindi Version) comprises 12 questions to assess psychological distress. The scale has good reliability and validity [18]. A cut-off score of <2 indicates that the caregiver is free from any psychiatric illness.

WHOQOL-BREF (Hindi version) was used to assess the quality of life and comprises four domains: physical, psychological, social, and environmental. The psychometric properties of this scale are comparable to those of the full version (WHOQOL-100) [19]. WHOQOL-BREF has good discriminant validity, concurrent validity, internal consistency, and test-retest reliability [19].

The WHOQOL-SRPB (Hindi version) consists of 32 items, each rated on a 5-point Likert scale. The questionnaire has eight facets related to spirituality, religiosity, and personal beliefs. The Cronbach alpha ranges from 0.77 to 0.95 (WHOQOL-SRPB Group, 2006), and for the complete instrument, it is excellent (0.93) [20]. The Cronbach alpha for each facet of the translated version came out to be 0.78 to 0.96.

Statistical analysis

Data were analyzed by using the statistical package SPSS 25.0 version software (IBM SPSS Statistics for Windows). The normalcy of the data was checked using the Kolmogorov-Smirnov test, and the variables were summarized using descriptive statistics. The caregiving experience at the two time points was assessed using a paired t-test or Wilcoxon signed rank test for normal and non-normal distribution data, respectively. Similarly, the knowledge of mental illness, family functioning, coping, social support, psychological distress, quality of life and spirituality, religiosity, and personal beliefs at the two time points were assessed using the paired t-test or Wilcoxon Signed Rank test. The two-sided p<0.05 was considered statistically significant.

## Results

Table 1 shows the characteristics of the patients and caregivers who were followed up. A majority of the patients were aged up to 35 years, while a majority of the caregivers were aged above 35 years. About half of the patients were male. A majority of the caregivers were married and employed, while a majority of the patients were not married and not employed. Among the caregivers, 28% (n = 14) were mothers, 24% (n = 12) were fathers, 20% (n = 10) were siblings, 20% (n = 10) were spouses, and 8% (n = 4) were children. A majority of the patients and caregivers belonged to nuclear families, urban backgrounds, and Hindu religion.

**Table 1:** Socio-demographic characteristics of the patients and caregivers Data shown as mean ± SD or n (%) where applicable.

Variable	Patient n (%)	Caregiver n (%)
Age (mean ± SD) years	33.50 ± 8.94	43.68 ± 15.64
18–25	11 (22)	9 (18)
26–35	20 (40)	9 (18)
36–45	11 (22)	6 (12)
46–55	8 (16)	12 (24)
>55		14 (28)
Gender		
Male	28 (56)	22 (44)
Female	22 (44)	28 (56)
Marital status		
Never married	29 (58)	13 (26)
Married	17 (34)	34 (68)
Separated	2 (4)	0 (0)
Divorced	1 (2)	1 (2)
Widowed	1 (2)	2 (4)
Religion		
Hindu	43 (86)	43 (86)
Muslim	5 (10)	5(10)
Sikh	2 (4)	2 (4)
Education		
Middle	3 (6)	3 (6)
Matric	7(14)	9 (18)
10+2	15 (30)	12 (24)
Graduate	21 (42)	21 (42)
Post-graduate	4 (8)	5 (10)
Occupation		
Professional	3 (6)	6 (12)
Skilled worker	1 (2)	8 (16)
Unskilled worker	1 (2)	3 (6)
Unemployed	28 (56)	4 (8)
Homemaker	11 (22)	13 (26)
Retired	0 (0)	7 (14)
Student	4 (8)	4 (8)
Business	2 (4)	4 (8)
Farmer	0 (0)	1 (2)
Area of residence		
Rural	3 (6)	3 (6)
Semi-urban	11 (22)	11 (22)
Urban	36 (72)	36 (72)

Table 2 shows the change in caregiving experience. Significant changes were observed in the domains of need for backup and dependency. Though changes were seen in overall negative and positive experiences of caregiving scores, the differences were not significant. The negative caregiving score was reduced in 31 caregivers (62%), did not change in 1 (2%), and increased in 18 (36%). The positive caregiving score increased in 25 caregivers (50%), did not change in 1 (2%), and was reduced in 23 caregivers (46%).

**Table 2:** Change in the caregiving experience Shown as mean ± SD. Comparisons were done using the Wilcoxon Signed Rank test (Z) for nonparametric data and paired t-test for parametric data. *Significant at p < 0.05. **Significant at p <0.01.

Caregiving experience	Baseline (n = 50) mean ± SD	Follow-up (n = 50) mean ± SD	Wilcoxon signed ranks/paired t-test	p-Value
Negative domains				
Difficult behaviours	1.48 ± 0.81	1.31 ± 0.90	Z = −1.211	0.226
Negative symptoms	2.00 ± 1.00	2.03 ± 0.84	Z = −0.339	0.735
Stigma	0.98 ± 0.76	1.03 ± 0.92	Z = −0.071	0.943
Problems with services	1.01 ± 0.70	0.86 ± 0.77	Z = −1.250	0.211
Effects on family	0.94 ± 0.72	0.93 ± 0.71	Z = −0.016	0.987
Need for backup	1.45 ± 0.67	1.20 ± 0.59	t = 2.370	0.022*
Dependency	2.34 ± 0.81	1.91 ± 0.86	t = 3.064	0.004**
Loss	1.50 ± 0.72	1.35 ± 0.67	t = 1.410	0.165
Total negative score	74.28 ± 29.96	67.32 ± 31.68	Z = −1.756	0.079
Positive domains				
Positive personal experiences	1.65 ± 0.75	1.68 ± 0.79	t = 0.225	0.823
Good aspects of the relationship	2.29 ± 0.69	2.30 ± 0.87	t = 0.104	0.918
Total positive score	26.92 ± 8.27	27.14 ± 9.15	t = 0.139	0.890

Table 3 presents the changes in other aspects of caregivers. The knowledge of the caregivers did not change significantly. Among the family functioning domains, the scores on communication and effective involvement were reduced significantly as compared to the baseline. Among coping, emotion-focused distraction-positive coping reduced significantly. While social support did not change significantly at follow-up, the psychological distress as measured by the General Health Questionnaire-12 (GHQ-12) significantly reduced. All the quality of life domains showed improvement with time. Among the spiritual, religious, and personal beliefs, a significant increase was seen only in the hope and optimism and inner peace domains.

**Table 3:** Change in other psychosocial variables of the caregivers Shown as mean ± SD. Comparisons were done using the Wilcoxon Signed Rank test (Z) for nonparametric data and paired t-test for parametric data. *Significant at p < 0.05; **significant at p < 0.01; ***significant at p < 0.001.

Psychosocial variables	Pre (n = 50) mean ± SD	Post (n = 50) mean ± SD	Wilcoxon Signed Ranks/paired t-test	p-Value
Knowledge of mental illness score	1.42 ± 1.53	1.72 ± 1.63	Z = −1.160	0.246
Family functioning				
Problem-solving	1.45 ± 0.42	1.47 ± 0.38	Z = −0.458	0.647
Communication	1.84 ± 0.52	1.63 ± 0.46	Z = −2.990	0.003**
Roles	2.27 ± 0.48	2.18 ± 0.51	t = 1.107	0.274
Affective responsiveness	1.92 ± 0.72	1.81 ± 0.70	Z = −0.582	0.561
Affective involvement	1.88 ± 0.64	1.66 ± 0.51	Z = −2.107	0.035*
Behavioural control	2.17 ± 0.60	2.03 ± 0.55	t = 2.002	0.051
General functioning	1.62 ± 0.51	1.49 ± 0.50	Z = −1.327	0.184
Coping				
Problem focused				
Problem-solving	0.57 ± 0.19	0.51 ± 0.22	Z = −1.359	0.174
Emotion focused				
Distraction positive	0.40 ± 0.14	0.34 ± 0.16	Z = −2.055	0.040*
Distraction negative	0.12 ± 0.15	0.08 ± 0.11	Z = −1.863	0.063
Acceptance/redefinition	0.64 ± 0.14	0.62 ± 0.16	Z = −0.133	0.894
Religion/faith	0.40 ± 0.24	0.39 ± 0.22	Z = −0.333	0.739
Denial/blame	0.35 ± 0.21	0.30 ± 0.19	Z = −1.733	0.083
Problem and emotion focused				
Social support	0.57 ± 0.31	0.47 ± 0.23	Z = −1.482	0.138
Social support	51.56 ± 8.27	53.68 ± 8.82	t = 1.512	0.137
Psychological distress	3.38 ± 3.24	2.34 ± 3.08	Z = −2.107	0.035*
Quality of life				
Physical health	16.28 ± 2.43	17.50 ± 1.90	Z = −3.518	<0.001***
Psychological health	14.28 ± 2.93	15.42 ± 2.27	Z = −2.946	0.003**
Social relationships	15.00 ± 2.60	15.96 ± 2.33	Z = −2.176	0.030*
Environment	15.12 ± 2.95	16.18 ± 2.45	Z = −2.566	0.010*
Spiritual, religious, and personal beliefs				
Connectedness	3.26 ± 1.11	3.29 ± 1.04	Z = 0.001	0.999
Meaning in life	3.59 ± 0.74	3.75 ± 0.70	Z = −1.308	0.191
Awe and wonder	3.63 ± 0.69	3.80 ± 0.63	Z = −1.571	0.116
Wholeness and integration	3.61 ± 0.74	3.73 ± 0.67	Z = −0.918	0.359
Spiritual strength	3.60 ± 1.06	3.64 ± 0.99	Z = −0.508	0.611
Inner peace	3.34 ± 0.84	3.56 ± 0.76	Z = −2.002	0.045*
Hope and optimism	3.61 ± 0.91	3.88 ± 0.78	t = 2.213	0.032*
Faith	3.54 ± 0.87	3.65 ± 0.91	Z = −0.644	0.519

## Discussion

The aim of this study was to examine the change in caregiving experience in relation to other psychosocial variables in the caregiver. The study found that some aspects of the caregiving experience changed over the period. Primarily, caregivers’ need for backup reduced over the follow-up period, and so did patient dependency. This suggests that with time, caregivers become more adept at handling the caregiving role and addressing this role without help from others. It is possible that learning occurs, either by trial or through the suggestion of others, about how to deal with difficult situations experienced during the caregiving process. Other negative aspects of the caregiving experience also seemed to decrease minimally (though the differences were not significant), though stigma seemed to increase marginally (again, non-significant differences). The overall negative aspects of caregiving showed a trend-level decrease. The positive aspects of caregiving largely remained stable, suggesting that a greater duration of caregiving does not necessarily enhance a positive appraisal of caregiving. These novel findings should be replicated in the future.

Studies have tried to examine the stability versus change in burden over the illness course. Some studies have found that caregiver burden remains unchanged over a period of follow-up [21,22], whereas other researchers have found a significant reduction [23,24]. In caregiving experience, similar results were found in Hayes et al. [25], where no significant differences were found in almost all domains in negative caregiving experience scores. However, in that study, both the positive subscales improved.

The present study also found improvements in the quality of life of the caregivers and a reduction in psychological distress. Literature shows some findings with no significant improvement in caregiver quality of life. [26]. Distress reduction among caregivers may also reflect the adjustment and coping with the disorder in the patient. Poon et al. [26] found no significant change in caregiver distress. One should be cautious while inferring the change to attributes of the caregiving role only, and there may be many other interpersonal, intrapersonal, and situational factors that influence the appraisal of quality of life and distress.

In family functioning, communication and affective involvement improved over time. It is possible that, with time, the caregivers modulated their communication after introspection and harmonized the expression of their emotions with the patient. There is some evidence to suggest that unhealthy family functioning reduces with time, as reported by caregivers of patients with schizophrenia [27], and that expressed emotions and emotional overinvolvement reduce over a period of time [28]. Coping largely did not change, except for emotion-focused distraction-positive coping, which reduced somewhat. The coping scores also did not change significantly in Chadda et al. [21]. Social support for caregivers did not change significantly. Poon et al. [26] supported the results. The spiritual, religious, and personal beliefs also did not largely change, except for improvements in inner peace and hope and optimism. One could argue that with time, the caregiver might be getting greater personality clarity about not being despondent and looking at the future with some positive expectations. Hope seems to be an important aspect of a family’s coping with the impact of mental illness [29].

There are some implications for the present findings. The study makes it clearer for mental health professionals that time may reduce some of the aspects of caregiving and that the caring process makes the caregiver more adept at dealing with the negative aspects of the caregiving role. The mental health professionals may be clearer about 'spontaneous’ improvement in some aspects of caregiving, primarily patient dependency. Another aspect was that stigma did not seem to come down with involvement in treatment. Thus, stigma reduction measures at the community level still need to be continued. Additionally, knowledge of mental illness did not show a significant change over the period of time. The results also imply that family interventions should be provided to caregivers, as they impact long-term patient and caregiver outcomes. Indian families are collectivistic in nature and have a significant role to play in the care of schizophrenia patients. Family is, therefore, a locus for change, and understanding Indian families is a must for involving them in psychotherapy [30]. This will lead to better treatment of the patient in the community and more satisfying outcomes.

Some limitations of the present study need to be highlighted. The study was conducted among caregivers from a single centre, and the sample was drawn through purposive sampling. Hence, generalizations should be drawn with caution. The sample size was low, and there may not be enough power to detect some changes. Furthermore, follow-up was done for only one time period within a year, and the trajectories of changes in caregiving experience over longer time periods might have been different. The changes may also have been influenced by many intercurrent factors, which the present study has not been able to factor in due to the small sample size. Nonetheless, the present exploratory research discusses the caregiving experience in a longitudinal manner and informs on changes in some parameters of caregiver experience.

## Conclusions

To conclude, the study reports that patient dependency and caregiver need for backup reduce with time among caregivers of patients with schizophrenia. Overall, the negative aspects of caregiving for schizophrenia show a trend-level decline, while the positive aspects of caregiving largely remain stable. Among the caregivers, quality of life improves with time, and psychological distress decreases. Future studies can look at the longer-term changes in caregiving experiences and also whether changes in caregiving experiences differ across the gender of the caregiver, the relationship with the index patient, cultural determinants of change in caregiver experiences, and the influence of different aspects like caregiver personality and coping styles.
